# Possible Natural Hybridization of Two Morphologically Distinct Species of *Acropora* (Cnidaria, Scleractinia) in the Pacific: Fertilization and Larval Survival Rates

**DOI:** 10.1371/journal.pone.0056701

**Published:** 2013-02-14

**Authors:** Naoko Isomura, Kenji Iwao, Hironobu Fukami

**Affiliations:** 1 Department of Bioresources Engineering, Okinawa National College of Technology, Nago-City, Okinawa, Japan; 2 Akajima Marine Science Laboratory, Zamamison, Okinawa, Japan; 3 Department of Marine Biology and Environmental Science, Faculty of Agriculture, University of Miyazaki, Miyazaki, Japan; Aberystwyth University, United Kingdom

## Abstract

Natural hybridization of corals in the Indo-Pacific has been considered rather rare. However, field studies have observed many corals with intermediate interspecific or unusual morphologies. Given that the existence of F1 hybrids with intermediate interspecific morphologies has been proven in the Caribbean, hybrids may also inhabit the Indo-Pacific and occur more frequently than expected. In this study, we focused on two morphologically different species, *Acropora florida* and *A. intermedia*, and performed crossing experiments at Akajima Island, Japan. Results showed that these species could hybridize in both directions *via* eggs and sperm, but that fertilization rates significantly differed according to which species provided eggs. These results are similar to those reported from the Caribbean. Although all embryos developed normally to the planular larval stage, the developmental processes of some hybrid embryos were delayed by approximately 1 h compared with conspecific embryos, suggesting that fertilization occurred 1 h later in interspecific crosses than in intraspecific crosses. More successful hybridization could occur under conditions with low numbers of conspecific colonies. Additionally, a comparison of survival rates between hybrid and intraspecific larvae revealed that intra- and interspecific larvae produced from eggs of *A. florida* survived for significantly longer than those produced from eggs of *A. intermedia*. Considering these data, under specific conditions, hybrids can be expected to be produced and survive in nature in the Pacific. Furthermore, we identified one colony with intermediate morphology between *A. florida* and *A. intermedia* in the field. This colony was fertilized only by eggs of *A. florida*, with high fertilization rates, suggesting that this colony would be a hybrid of these two species and might be backcrossed.

## Introduction

Hybridization occurs in many animals and plants. Especially in plants, reticulate evolution, in which hybridization and speciation are repeated in evolutionary time, has been reported [1], [2]. In marine animals, several kinds of hybridization have been reviewed [3], [4]. Veron [5] first proposed the hypothesis of reticulate evolution in zooxanthellate scleractinian corals (hereinafter referred to as corals). Many species of coral spawn synchronously on the same night and time in early summer (i.e., mass spawning) [6], [7], [8]. In the Indo-Pacific, such mass spawning of the most abundant corals, genus *Acropora*, might cause hybridization because large quantities of their eggs and sperm mix in mass spawning events. In fact, hybridization of *Acropora* species has been proven in crossing experiments [9], [10], [11], [12], [13]. In addition, molecular phylogenetic analyses have revealed that hybridization occurs only among genetically closely related species [11], suggesting the presence of the repeated hybridization of *Acropora*, i.e., reticulate evolution. Furthermore, study of *Acropora* chromosomes also supported a hypothesis of reticulate evolution [14]. Hybridization has been suggested also for other corals, including *Platygyra* [15], [10], *Montipora* [10], and the Caribbean massive corals, the *Montastraea annularis* (Ellis and Solander, 1786) complex. Particularly detailed reproductive, genetic, and regional analyses of the *M. annularis* complex have been performed [16], [17], [18], [19], [20], [21].

In contrast to diverse Indo-Pacific *Acropora*, only three species of *Acropora* inhabit the Caribbean: *A. palmata* (Lamarck, 1816), *A. cervicornis* (Lamarck, 1816), and *A. prolifera* (Lamarck, 1816). *Acropora prolifera* has been recognized as a hybrid between *A. palmata* and *A. cervicornis*, based on genetic [22], [23] and reproductive [24] studies. This hybrid species, *A. prolifera*, has been known to be nearly sterile, suggesting that reticulate evolution with repeating hybridization and speciation may not occur in the Caribbean [23]. Vollmer and Palumbi [23] proposed that no reticulate evolution occurs in the Indo-Pacific, either, because sterile F1 hybrids may have been produced in the Indo-Pacific, as in the Caribbean. Opposing this suggestion, Miller and van Oppen [25] strongly insisted on the possibility of reticulate evolution in the Indo-Pacific. Recently, Willis et al. [13] reviewed the hybridization of corals and suggested that hybridization may occur in the Indo-Pacific only in peripheral regions of a coral species' distribution, but not in main tropical and subtropical reefs, although Wei et al. [26] reported no hybridization of *Acropora* species in Taiwan, which is a marginal habitat of corals. Thus, since Willis et al. [13], hybridization in the Indo-Pacific, especially of *Acropora*, has been believed to be quite rare.

Compared with hybrid studies of Caribbean corals, a paucity of species-specific data on the hybridization and on the survival rates of hybrid larvae exists for the Indo-Pacific because of the high number (>100) of resident *Acropora* species [27]. Detailed analyses similar to those performed on Caribbean *Acropora* [24] and *Montastraea* [21] should be performed in the Indo-Pacific. In addition, Indo-Pacific hybrid morphologies remain largely unknown, and have been reported only for *A. millepora* (Ehrenberg, 1834) × *A. pulchra* (Brook, 1891) [13]. Furthermore, to date, insufficient data are available to judge the occurrence of natural hybridization in the Indo-Pacific. Willis et al. [13] performed sperm selection experiments, mixing intra- and interspecific sperm against intraspecific eggs, and indicated that hybridization does not occur under conditions in which intraspecific sperm exists. However, they also suggested that hybridization may occur in regions with a low number of conspecific colonies.

Around Okinawa, Japan, coral reefs are well developed and more than 300 species have been reported [28]. However, since the mass bleaching event of 1998, a large amount of corals in Japan had decreased dramatically, due to repeated bleaching and outbreaks of crown-of-thrown sea stars [29], [30], [31], [32]. Around Akajima Island, where our study was performed, the number of *Acropora* colonies also decreased [32], [33]. The condition may be assumed to be the peripheral region of distribution of some *Acropora* species, in which low opportunity of intraspecific fertilization may increase opportunity of interspecific fertilization.

**Figure 1:**
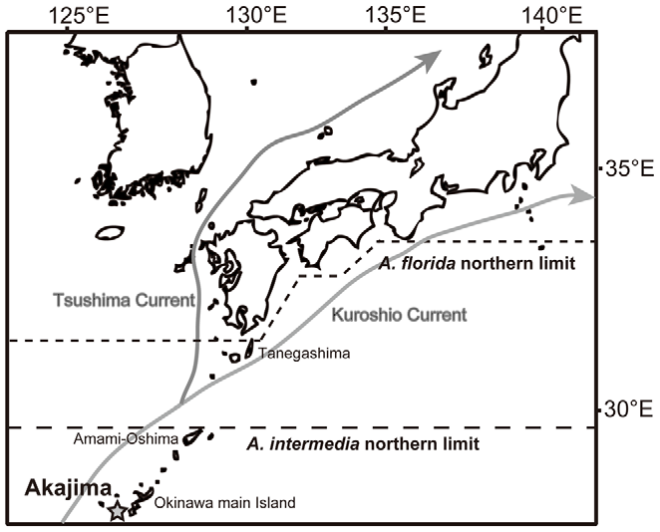
Map of Japan showing the sampling site. Star shows the location of Akajima in Japan. Dash lines show northern limits of distribution [27] of two species, *Acropora intermedia* and *A. florida*.

In this study, we focused on two morphologically different species, *A. florida* (Dana, 1846) and *A. intermedia* (Brook, 1891) (previously recognized as *A. nobilis* (Dana, 1846), see [27] in detail). These species are very common and sympatric in the Indo-Pacific (see Fig. 1 for the species distribution), and Hatta et al. [11] reported their hybridization with high (>70%) fertilization rates. These species are useful in hybridization studies because the parent species clearly differ morphologically, with *A. florida* being hispidose (bottle-brush–like) and *A. intermedia* being arborescent (long branch-like), meaning that the morphologies of probable F1 hybrids would be easily detectable. These two species also belong to a genetically closely related group (uncorrected p distance 0–2.5% in the mini-collagen gene [11]), suggesting the high possibility of hybridization in the past [11]. Thus, to examine the possibility of natural hybridization, we investigated the fertilization rates, embryonic development, and larval survivorship of *A. florida*, *A. intermedia*, and hybrids of these two species.

## Materials and Methods

### Crossing experiments

Crossing experiments were conducted on 6 June 2007 and 10 June 2012. For crossing experiments, five colonies of *A. florida* and seven colonies of *A. intermedia* in 2007, and five colonies of *A. florida*, four colonies of *A. intermedia*, and one colony of *A.* sp. “int-flo” (see below) in 2012 were collected from reefs around Akajima Island Okinawa, Japan (Fig. 1; 26°12′N, 127°17′E) with permission to Akajima Marine Science Laboratory from the Okinawa Prefectural Government (# 24–17). After collection, colonies were kept in the sea (2007) or in a water tank with running seawater (2012) until spawning. When settings of bundles on polyp mouths were observed, colonies were transferred into individual buckets. Gamete bundles were then collected from individual colonies using Pasteur pipettes and allowed to break apart in a small volume of filtrated seawater (10 *µ*m pore size) to yield free eggs and sperm suspensions. These experiments were conducted in a room maintained at 26°C. Eggs were washed twice with filtrated seawater, and sperm suspensions were diluted to adjust their concentrations. Eggs and sperm collected from individual colonies were then mixed in pairwise combinations. Two hundred to three hundred eggs were mixed with sperm in 60-ml vials within 2 h after spawning. Final sperm concentrations were 0.5–2×10^6^/ml, which is an effective fertilization concentration for *Acropora* [10]. The numbers of fertilized and unfertilized eggs were scored at the 16-cell/morula stage 4–5 h after gamete mixing. Also, to compare differences in the development process between intra- and interspecific crosses, the developmental stage of fertilized eggs was observed every 1–2 h after insemination. Self-fertilization experiments were also conducted at the same time.

Average fertilization rates for intra- and interspecific crosses were analyzed statistically by Mann–Whitney *U-*tests and Kruskal–Wallis tests, and all pairwise multiple comparisons were performed by the Steel-Dwass method.

### Measurement of planular survivorship

In 2012, thirty-five hours after the start of the experiment, we transferred 20 planular larvae from each crossing combination into each of four Petri dishes. Planulae in the Petri dishes were counted and their condition was checked every 24 h for 30 days. The mean number of planulae alive in the four replicates was considered to represent planular survivorship. We calculated a survival curve for planular larvae based on the Kaplan–Meier estimator to evaluate larval survivorship [34]. To compare survivorship between two colonies, we determined the distribution of each. If the distribution was exponential, we used a Cox–Mantel test. If distribution was Weibull, we used a Wilcoxon test. If distribution was neither exponential nor Weibull, we used a log-rank test [35].

### Species identification and morphological analyses

**Figure 2:**
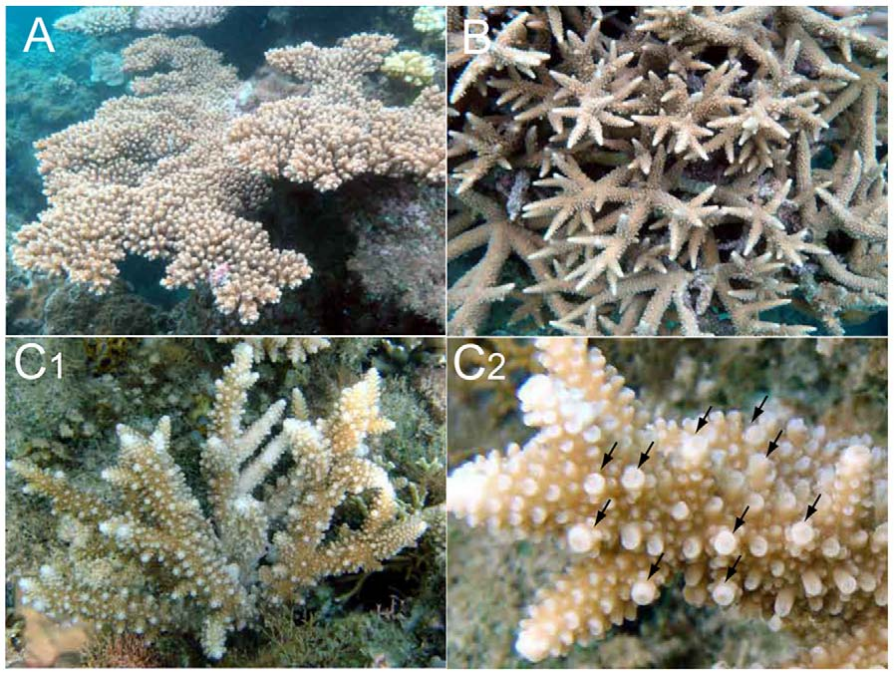
The *Acropora* species used for crossing experiments. A) *A. florida*, B) *A. intermedia*, and C) *A*. sp. “int-flo” for whole colonies (subscripted with “1”) and close-ups of branches (Subscripted with “2”). Arrows show secondary short branches or incipient axial corallites.

**Figure 3:**
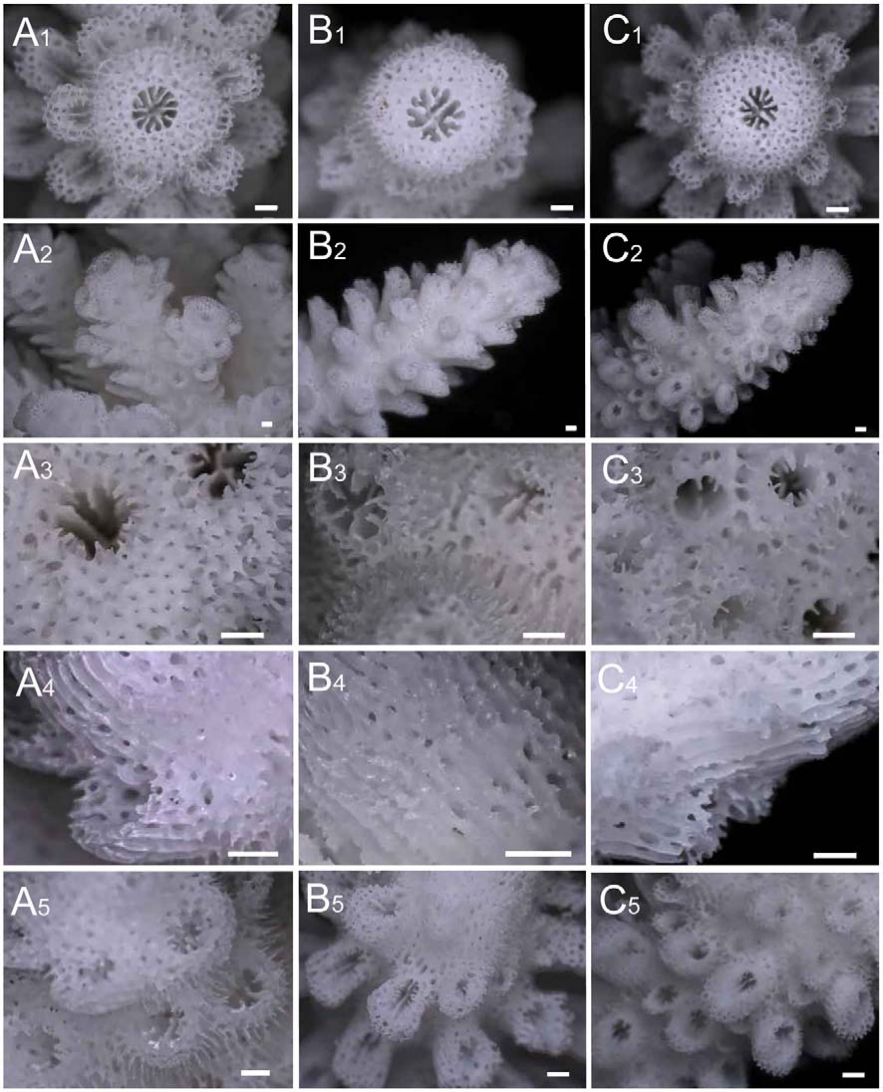
Macro-scale skeletal morphologies of the specimens used for crossing experiments. A) *Acropora florida*, B) *A. intermedia*, and C) *A*. sp. “int-flo.” Axial corallites (subscripted with “1”), lateral side views of the branches (subscripted with “2”), coenosteum and immersed radial corallites (subscripted with “3”), lateral side views of axial corallites (subscripted with “4”), and structures of the radial corallites (subscripted with “5”) are shown. The sample numbers of each species are AK63 for *A. florida*, AK50 for *A. intermedia*, and AK70 for “int-flo.” All bars show 500 µm.

All specimens used in crossing experiments were bleached for exact species identification and morphological analysis. Skeletal morphologies were observed using a digital microscope (Keyence). Species identification was mainly performed following literatures [27], [36], [37]. Based on taxonomic references, all specimens except one were identified as *A. florida* or *A. intermedia* (Fig. 2A, 2B). For one colony, species identification was problematic for the following reasons: the colony shape was arborescent or nearly caespitose (Fig. 2C) and the colony appeared superficially to be *A. intermedia* or the branching type of *A. florida*. The axial corallites were large and dome-shaped (Fig. 3C_1_), which are also similar to *A. florida* and *A. intermedia* (Fig. 3A_1_, 3B_1_). The radial corallites (Fig. 3A_2_, 3B_2_, 3C_2_) were dimorphic and had long, tubular shapes with dimidiate or oblique openings [27], which suggested *A. intermedia*, as those of *A. florida* are appressed tubular shapes with round openings. Notably, this colony had many short secondary branches as well as incipient axial corallites (Figs. 2C_2_, 3C_1_), but did not form the typical hispidose shape of *A. florida*. The microstructure of this colony, such as the shapes of the radial corallites and spinules of the coenosteum (Fig. 3C_4–5_) were similar to those of *A. intermedia* (Fig. 3B_4–5_). Thus, this colony was intermediate in morphology, between *A. florida* and *A. intermedia*, and it was impossible to identify with certainty. Therefore, in this study, we named this morphology *A*. sp. “int-flo” (hereinafter, “int-flo”).

## Results

### Fertilization rate

Spawning of our specimens was observed on 6 July 2007 and 10 July 2012. In 2007, bundle setting, during which egg-sperm bundles were set on the polyp mouths, started in *A. florida* and *A. intermedia* from 20∶10 until 20∶30 (local time in Okinawa, Japan), with sunset occurring at 19∶21. Spawning started at 22∶30 (189 min after sunset) in *A. florida* and at 22∶40–23∶00 (199–219 min after sunset) in *A. intermedia*. In 2012, bundle setting started in *A. florida*, *A. intermedia*, and “int-flo” from 20∶15 until 20∶45, with sunset occurring at 19∶23. Spawning started at 21∶45–21∶50 (142–147 min after sunset) in *A. florida*, and at 22∶15–22∶30 (172–187 min after sunset) in *A. intermedia* and “int-flo.” Thus, the spawning time of *A. florida* was 10–40 min earlier than those of other species. The spawning of all species continued for 10–20 min in 2007 and 2012.

**Figure 4:**
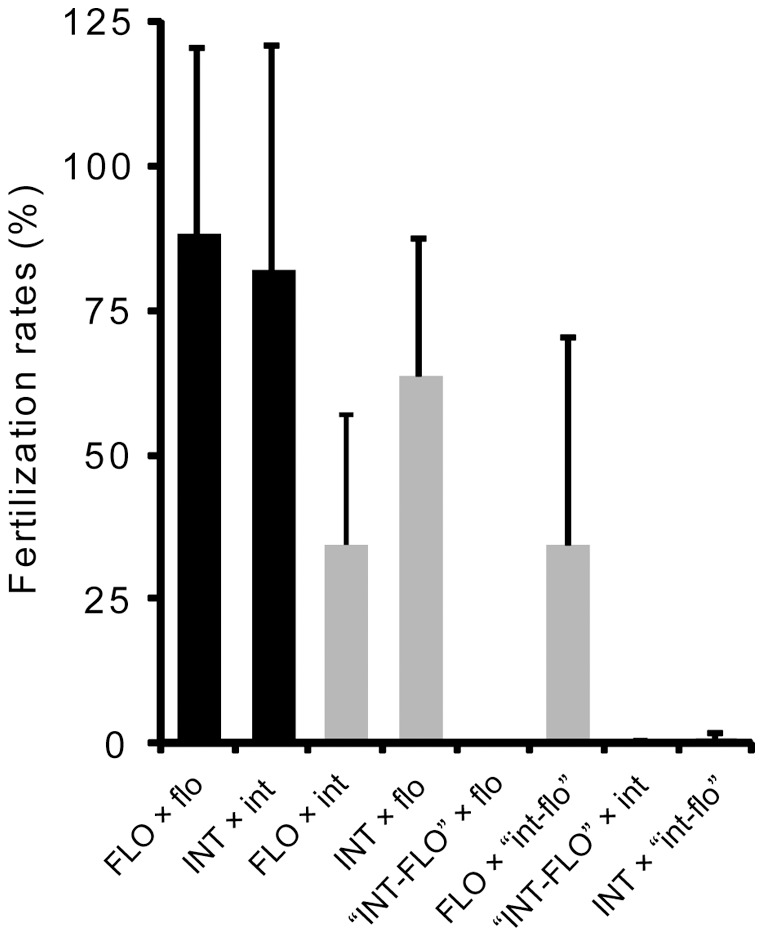
Average fertilization rates in all crossing experiments performed in 2007 and 2012. Intraspecific crosses are shown in black, and interspecific crosses are shown in gray. flo, *A. florida*; int, *A. intermedia*; “int-flo”, *A*. sp. “int-flo.” Uppercase text indicates eggs and lowercase text indicates sperm. Error bars represent standard deviations.

**Figure 5:**
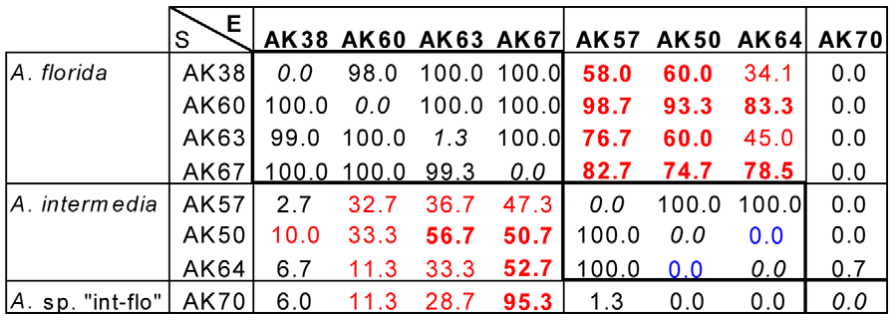
An example of fertilization in the crossing experiments of 10 June 2012. Values are shown as percentages. Interspecific crosses with fertilization rates >10% are shown in red (>50% in bold). Low fertilization rates within species are shown in bold italics. Note that rates of self-fertilization were very low. Sperm concentrations were 0.6–1.5×10^6^. Colony numbers are shown in the left column (AK plus numbers). E, eggs; S, sperm.

Fertilization rates are summarized in Table 1 and Fig. 4. Figure 5 shows examples of the crosses performed in 2012. Self-fertilization was not observed (0%) in 19/20 colonies, but was noted in one colony (#AK63) of *A. florida*, where the rate of self-fertilization was quite low (1.3%; see Fig. 5). Intraspecific crosses of the two species (*A. florida* and *A. intermedia*) showed high fertilization rates (82.1–88.1% on average; Table 1). Although the intraspecific crosses between two colonies each of *A. florida* and *A. intermedia* had 0% fertilization rates, these colonies were fertilized at nearly 100% with other colonies within species (see Fig. 5 for crosses between #AK50 and #AK64 of *A. intermedia*), suggesting that these colonies might have been clones or genetically similar [35].

**Table 1:** Fertilization rates of intra- and interspecific crosses performed in 2007 and 2012.

	I	II	III	IV	V	VI	VII	VIII
No. of colonies	7	6	11, 11	8, 7	1, 5	5, 1	1, 4	4, 1
No. of crosses	18	12	34	22	5	5	4	4
Ave FR (SD)	88.1 (32.1)	82.1 (38.4)	34.2 (22.5)	63.5 (24.1)	0.1 (0.3)	34.5 (35.7)	0.2 (0.4)	0.8 (1.0)
Min–Max FR	0–100	0–100	1.5–93.0	17.3–98.7	0–0.6	6.0–95.3	0–0.7	0–2.0
*P*<0.01	III, IV	nd	IV, V	V	III, IV	nd	nd	nd
*P*<0.05	V	III, IV	VII, VIII	VII, VIII	I	nd	III, IV	IV

Roman numbers mean combinations of crosses; **I** for *A. florida* eggs × *A. florida* sperm, **II** for *A. intermedia* eggs × *A. intermedia* sperm, **III** for *A. florida* eggs × *A. intermedia* sperm, **IV** for *A. intermedia* eggs ×*A. florida* sperm, **V** for *A.* sp. “int-flo” eggs × *A. florida* sperm, **VI** for *A. florida* eggs × *A.* sp. “int-flo” sperm, **VII** for *A.* sp. “int-flo” eggs × *A. intermedia* sperm, **VIII** for *A. intermedia* eggs × *A.* sp. “int-flo” sperm. For intraspecific crosses (I, II), total number of colonies we used is shown, and for interspecific crosses (III–VIII), total colony numbers for eggs and for sperm are shown respectively. Values of average (Ave), and minimum (Min) and maximum (Max) fertilization rates (FR) are shown by %. Combinations (I–VIII) that significantly differed by all pairwise multiple comparisons (see Results) were shown in bottom. nd means “not detected”. SD means standard deviation.

Fertilization rates of interspecific crosses were recorded between *A. florida* and *A. intermedia*, and between *A. florida* and “int-flo” (Table 1, Figs. 4, 5). Notably, the fertilization rates differed with combinations of eggs and sperm. Crosses of *A. florida* eggs × *A. intermedia* sperm showed an average fertilization rate of 34.2%, but reciprocal crosses showed an average fertilization rate of 63.5%. Similarly, the fertilization rate of *A. florida* eggs × “int-flo” sperm was 34.5% on average, but 0% in reciprocal crosses. In the crosses shown in Fig. 4, the fertilization rates of most combinations of *A. intermedia* eggs × *A. florida* sperm exceeded 50% (maximum, 98.7%). In turn, the reciprocal crosses showed fertilization rates of <50% in most combinations. Crosses between *A. florida* and “int-flo” showed highly variable (6.0–95.3%) fertilization rates.

The average fertilization rates of intraspecific crosses between *A. florida* and *A. intermedia* did not differ significantly (Mann–Whitney *U-*test, *Z* = 2.57, *p* = 0.14), whereas the fertilization rates of interspecific crosses among combinations differed significantly (Kruskal–Wallis test, *χ^2^* = 44.30, *p* = 0.00). Multiple comparisons (Table 1) revealed significant differences between intra- and interspecific crosses using *A. florida* and *A. intermedia*; *A. florida* eggs × *A. florida* sperm and *A. florida* eggs × *A. intermedia* sperm (*χ^2^* = 4.59, *p* = 0.00), *A. florida* eggs × *A. florida* sperm and *A. intermedia* eggs × *A. florida* sperm (*χ^2^* = 4.09, *p* = 0.00), *A. intermedia* eggs × *A. intermedia* sperm and *A. florida* eggs × *A. intermedia* sperm (*χ^2^* = 3.40, *p* = 0.01), *A. intermedia* eggs × *A. intermedia* sperm and *A. intermedia* eggs × *A. florida* sperm (*χ^2^* = 3.03, *p* = 0.04). In addition, significant difference was found between *A. florida* eggs × *A. intermedia* sperm and the reciprocal cross (*χ^2^* = 3.95, *p* = 0.00). For the crosses using “int-flo”, *A. florida* eggs × “int-flo” sperm did not differ significantly against any other crosses although total number of crosses of this combination was too small (Table 1).

### Development

All embryos of each intraspecific cross developed normally, as shown in [38], whereas the embryos of interspecific crosses were at different developmental stages at the same time for every cross, i.e., the two-, four-, and eight-cell stages were mixed within a cross. For example, 3 h after insemination, all embryos in the intraspecific crosses had reached the eight-cell stage, while 2–8 cells were observed simultaneously in the interspecific crosses. At 4–4.5 h after insemination, all embryos of intraspecific crosses had reached the morula stage, but only after 5.5 h had all embryos of interspecific crosses reached this stage. Nevertheless, all embryos in all crosses became planular larvae 24 h after insemination.

### Planular survivorship

None of the larval survival curves showed exponential or Weibull distributions, so we used log-rank tests to compare survivorship among crosses. However, we recalculated the significance levels based on the Bonferroni correction (*α* = 0.05/6 = 0.0083) when comparing the survival curves of more than four crosses.

**Figure 6:**
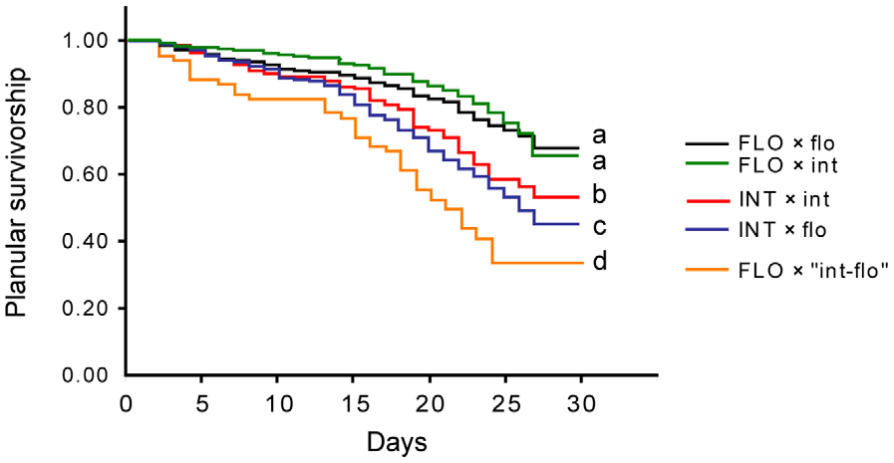
The larval survival curves of mating pairs. flo, *A. florida*; int, *A. intermedia*; “int-flo,” *A*. sp. “int-flo.” Uppercase text indicates eggs and lowercase text indicates sperm. Survival curves with different symbols (a–d) show significant differences in survivorship.

Figure 6 shows the survival curves of larvae resulting from intra- and interspecific crosses. Two intra- and interspecific crosses (*A. florida* eggs × *A. florida* sperm and *A. florida* eggs × *A. intermedia* sperm) showed the highest survivorship, and they showed no significant difference in larval survivorship (*χ^2^* = 0.13, *p* = 0.17). The interspecific cross of *A. intermedia* eggs × *A. florida* sperm showed significantly lower survivorship than did intraspecific cross of *A. intermedia* eggs × *A. intermedia* sperm (*χ^2^* = 4.08, *p* = 0.04). Crosses of *A. florida* eggs × “int-flo” sperm showed significantly lower survivorship than did those of all other crosses (*p* = 0.00–0.02).

## Discussion

### Hybridization of *Acropora*

In this study, fertilization rates between *A. florida* and *A. intermedia* differed significantly between combinations of eggs and sperm (34.2% and 63.5% on average). The fertilization rates of *A. intermedia* eggs × *A. florida* sperm were higher than those of the reciprocal crosses. This result is very similar to that of a crossing experiment between *A. florida* and *A. intermedia* in 1998 reported by Hatta et al. [11]. The similar data obtained in 1998 [11], 2007, and 2012 (this study) suggest that the fertilization rates of these two species usually differ between combinations of eggs and sperm. This pattern is also similar to that of Caribbean *Acropora* species, in which the fertilization rates of two species, *A. palmata* and *A. cervicornis*, differ between combinations of eggs and sperm [24].

All embryos in the intraspecific crosses developed normally, as shown in [38], whereas gametes from interspecific crosses developed unevenly (see Results). Overall, many (not all) embryos of interspecific crosses were developmentally delayed by 30–60 min compared with those of intraspecific crosses, although all embryos of intra- and interspecific crosses had developed to the morula stage by 5 h after insemination. Considering that embryos of interspecific crosses developed normally (same as intraspecific embryos) after two cells stage, one main reason of delayed development of interspecific crosses could be a delay of egg-sperm fertilization. The reasons for delayed fertilization remain unclear, but it may be a survival mechanism by hybridization for *Acropora* species in severe conditions, such as those under which depleted conspecific populations may be found; i.e., when few gametes of conspecific colonies are present for fertilization, hybridization with gametes of sympatric particular species may occur. Morita et al. [39] showed that the sperm did not respond to the eggs among species that cannot hybridize. At present, the response of sperm to eggs among species that can hybridize remains unknown, but this work may provide clues to understanding the underlying mechanism.

The tendencies of planular survivorship were reversed from those of fertilization rates. Planular survivorship between *A. florida* eggs × *A. intermedia* sperm (34.2% fertilization) was as high as that of intraspecific crosses of *A. florida*, whereas planular survivorship between *A. intermedia* eggs × *A. florida* sperm (63.5% fertilization) was lower than those of *A. florida* eggs × *A. intermedia* sperm and intraspecific crosses of *A. florida* (Fig. 6). In acroporids, almost all planulae show settlement 5–8 days after fertilization [40], [41] and >50% of planulae die by 30 days [40]. Meanwhile, some planulae remain competent to settle 30 days after fertilization [42]. In this study, of twenty planulae, eight to twelve planulae showed normal metamorphosis and settlement after 8 days in every crossing experiment when there was conditioned plate to be able to be settled by planulae. More detailed studies of settlement are required, but hybrid planulae may have high survival potential during the planular stage because their survivorship was higher than that of intraspecific crosses at 5–8 days. In scallops (among marine invertebrates; [43]), higher growth and survival have been reported for hybrids than for intraspecific crosses in early-life stages. Similarly, the larvae of *A. florida* × *A. intermedia* hybrids may have greater potential for survival than do larvae produced from intraspecific crosses. Thus, hybridization does not appear to negatively affect early life stages, and hybrids can survive at least these stages.

### Possibility of hybrids

In this study, we identified an “int-flo” colony with an intermediate morphology between *A. florida* and *A. intermedia*. The species identification of this colony was problematic due to its unusual morphological characters. Although we did not focus on taxonomy in this study, we will discuss briefly some taxonomic characters of this morphotype for future studies. When we collected the colony in the field, we first tentatively identified it as a morphological variant of *A. intermedia* or *A. florida*, due to the colony shape with thick branches and its sympatry with these species. However, at least two skeletal morphological characters (radial corallites, and numerous sub-branches) differed from the typical skeletal morphology of *A. intermedia* or *A. florida* (see method). In total, “int-flo” differed morphologically from *A. intermedia* and *A. florida*, but would be phylogenetically closely related to both of these species due to the shared characteristic of dome-shaped large axial corallites (Fig. 3A_1_, B_1_, C_1_). In addition, the peculiar characteristics of numerous sub-branches and incipient axial corallites on a main branch have also been reported in a few species; e.g., *A. samoensis* (Brook, 1891) (senior synonym of *A. wallaceae* Veron, 1990), shown in [27], [37], or the Indian and Red Sea species *A. forskali* (Ehrenberg, 1834) and *A. pharaonis* (Milne Edwards & Haime, 1860) [44]. These species, however, differ morphologically and ecologically from “int-flo” in some aspects (i.e., radial corallite structure, colony shape, and species distribution). At present, we cannot determine whether “int-flo” is an undescribed species or a morphological variant of a known species. Morphological analyses and taxonomical studies using more samples of this morph may yield a more precise identification of “int-flo.”

**Figure 7:**
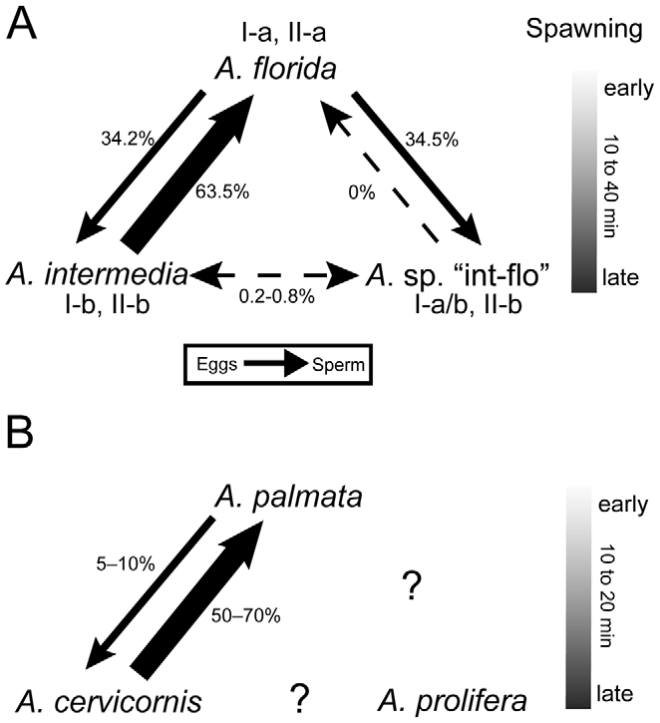
Summary of fertilization rates in crossing experiments, spawning times, and morphological characteristics. A) this study and B) the Caribbean. All data of the Caribbean were referred from [24]. Arrows show eggs fertilized by sperm (dash, nearly 0%; thin line, <50%; thick line, >50%). Question marks mean no data available. Roman numerals and letters show the following morphological characters; I, colony form (a, hispidose; b, arborescent); II, radial corallite structure (a, appressed tubular with round opening; b, dimorphic, long tubular with dimidiate or oblique opening).

However, considering the difficulty of species identification, the morphological similarities to *A. florida* and *A. intermedia*, and its hybridization with eggs of *A. florida* (summarized in Fig. 7), “int-flo” is highly likely to be a hybrid produced by combinations of *A. florida* and *A. intermedia*. Richards et al. [45] also suggested that rare *Acropora* species in the Indo-Pacific are probably hybrids. As mentioned above, “int-flo” had a mixture of morphological patterns of *A. florida* and *A. intermedia*. Such mixed morphologies have been reported in the *A. humilis* group (*A. humilis*, *A. gemmifera*, *A. monticulosa*, and *A. digitifera*) classified by [27]. Based on morphological, reproductive, and genetic analyses of the *A. humilis* group, Wolstenholme [46] suggested that two morphs, “mont-hum” (a mixture of *A. monticulosa* and *A. humilis*) and “terete-mont” (an unusual shape of *A. monticulosa*), had hybrid origins derived from *A. humilis* and *A. monticulosa*. Further molecular analyses of “int-flo” or the growth of the hybrids produced in this study to mature colonies are necessary to clarify the possible hybrid status of “int-flo”.

In the Caribbean, fertilization rates of *A. palmata* eggs × *A. cervicornis* sperm were 5–10% on average, while those of the reciprocal crosses were 50–70%. In addition, spawning time sometimes overlaps in these two Caribbean species, but *A. palmata* spawns about 10 to 20 min earlier than *A. cervicornis*, a pattern similar to that observed for *A. florida* and *A. intermedia*; i.e., *A. florida* spawned 10–40 min earlier than did *A. intermedia* (Fig. 7). Nevertheless, in the Caribbean, the colony shape of the F1 hybrid *A. prolifera* differs depending on which species provides eggs, suggesting that natural hybridization can occur in both directions, despite differences in spawning timing and low (5–10%) fertilization rates in one direction. Thus, considering data from Caribbean *Acropora*, it is highly possible that *A. florida* and *A. intermedia* hybridize in the field, at least around Akajima, Okinawa Japan. “Int-flo,” which had intermediate morphology between *A. florida* and *A. intermedia*, is likely to be a hybrid.

Although this study did not provide direct evidence of natural *Acropora* hybrids in the Pacific, the larval survival experiments inspired us to examine the role of hybrids. Survivorship rates were higher in intraspecific larvae of *A. florida* and hybrid larvae of *A. florida* eggs × *A. intermedia* sperm than in intraspecific *A. intermedia* larvae and hybrid larvae of *A. intermedia* eggs × *A. florida* sperm, suggesting that the eggs of *A. florida* induce larval survivorship more effectively than do those of *A. intermedia*, even though the larvae were hybrids (Fig. 6). Nevertheless, the survivorship of larvae from *A. florida* eggs × “int-flo” sperm was the lowest among all comparisons. This finding suggests that crosses of *A. florida* eggs × “int-flo” sperm might be backcrosses with reduced larval survivorship.

Willis et al. [13] suggested that the hybridization of corals may be more frequent at peripheral boundaries of species' ranges, which means lower number of conspecific colonies. A decrease in coral may cause a higher incidence and/or survivorship of hybrids. Around Akajima Island, a large amount of corals had been decimated by the outbreak of the crown-of-thorns starfish, *Acanthaster planci* during recent 2001 to 2006 [47], or coral bleaching there since 1998 [33]. These situations might increase hybridization rates, and “int-flo” may be a product of this process. More field research into the existence of “int-flo” and morphological, reproductive, and genetic comparisons of the artificial hybrids of *A. florida* and *A. intermedia* would provide us with more critical data in the near future.

In conclusion, to prove directly the existence of natural hybrids in the Pacific, growing hybrids produced in crossing experiments to adult colonies is absolutely necessary to examine their morphologies and reproductive capacities. To date, we have maintained the hybrids produced from the *A. florida* × *A. intermedia* crosses. In the near future, we hope to present further data to increase the understanding of *Acropora* hybridization.
